# Associations between e-cigarette access and smoking and drinking behaviours in teenagers

**DOI:** 10.1186/s12889-015-1618-4

**Published:** 2015-03-31

**Authors:** Karen Hughes, Mark A Bellis, Katherine A Hardcastle, Philip McHale, Andrew Bennett, Robin Ireland, Kate Pike

**Affiliations:** Centre for Public Health, Liverpool John Moores University, 15-21 Webster Street, L3 2ET Liverpool, UK; Public Health Wales, Hadyn Ellis Building, Maindy Road, CF24 4HQ Cardiff, UK; Health Equalities Group, 2nd floor, 151 Dale Street, L2 2JH Liverpool, UK; Trading Standards North West, New Town House, Buttermarket Street, WA1 2NH Warrington, UK

**Keywords:** Electronic cigarettes, Adolescents, Smoking, Alcohol use

## Abstract

**Background:**

Public health concerns regarding e-cigarettes and debate on appropriate regulatory responses are focusing on the need to prevent child access to these devices. However, little is currently known about the characteristics of those young people that are accessing e-cigarettes.

**Methods:**

Using a cross-sectional survey of 14-17 year old school students in North West England (n = 16,193) we examined associations between e-cigarette access and demographics, conventional smoking behaviours, alcohol consumption, and methods of accessing cigarettes and alcohol. Access to e-cigarettes was identified through a question asking students if they had ever tried or purchased e-cigarettes.

**Results:**

One in five participants reported having accessed e-cigarettes (19.2%). Prevalence was highest among smokers (rising to 75.8% in those smoking >5 per day), although 15.8% of teenagers that had accessed e-cigarettes had never smoked conventional cigarettes (v.13.6% being ex-smokers). E-cigarette access was independently associated with male gender, having parents/guardians that smoke and students’ alcohol use. Compared with non-drinkers, teenagers that drank alcohol at least weekly and binge drank were more likely to have accessed e-cigarettes (adjusted odds ratio [AOR] 1.89, P < 0.001), with this association particularly strong among never-smokers (AOR 4.59, P < 0.001). Among drinkers, e-cigarette access was related to: drinking to get drunk, alcohol-related violence, consumption of spirits; self-purchase of alcohol from shops or supermarkets; and accessing alcohol by recruiting adult proxy purchasers outside shops.

**Conclusions:**

There is an urgent need for controls on the promotion and sale of e-cigarettes to children. Findings suggest that e-cigarettes are being accessed by teenagers more for experimentation than smoking cessation. Those most likely to access e-cigarettes may already be familiar with illicit methods of accessing age-restricted substances.

## Background

Rapid growth in the marketing and use of e-cigarettes is generating widespread public health debate across the globe. Designed to provide a comparable smoking experience to conventional cigarettes, e-cigarettes are battery-powered nicotine delivery devices that provide doses of nicotine through an aerosol, typically combined with flavouring and driven by a component such as propylene glycol [1,2]. Unlike many nicotine replacement therapies, e-cigarettes are often marketed to smokers as healthier alternatives to conventional tobacco products, rather than as aids to ending nicotine dependence [3]. Proponents stress their harm reduction benefits in moving smokers away from the damaging toxins in conventional tobacco products, and their potential in helping them quit smoking altogether [4,5]. However, opponents highlight their unknown quality, safety and efficacy; their deleterious impact on the ‘no smoking’ health message; and their potential to harm children, including as a possible gateway to cigarette smoking [2,4,6]. Thus, despite growing consensus among health professionals that e-cigarettes are a less damaging delivery mechanism for nicotine than conventional tobacco products [6], a lack of regulation governing them in many countries means that an addictive drug can be promoted to, and cheaply accessed by, children [7]. However, the extent of children’s access to e-cigarettes and the characteristics of those that access them remain poorly defined.

Research on e-cigarette use in children is only just emerging, yet studies in several countries are showing their rapid penetration into adolescent markets. In the USA, studies show that lifetime e-cigarette use in grades 6-12 school students (~age 11-18) more than doubled from 3.3% in 2011 to 6.8% in 2012 [1]. Whilst most e-cigarette users in the 2012 survey were current smokers, 9.3% had never smoked a conventional cigarette. In France, a 2012 study of Parisian school students aged 12-19 years found that 8.1% had tried e-cigarettes, with prevalence ranging from 4.4% of non-smokers up to 33.4% of regular smokers [8]. In Korea, prevalence of ever using e-cigarettes in 13-18 year olds was found to have increased almost 20-fold between 2008 and 2011, from 0.5% to 9.4%, with 1.4% of e-cigarette users in 2011 having never smoked cigarettes [9].

In the UK, the marketing and sale of e-cigarettes has increased substantially over recent years [3,10], with 1.3 million people estimated to use them in 2013 [11]. A survey in 2013 found that two thirds of 11-18 year olds had heard of e-cigarettes, and that 5% of those who had heard of them had tried them [12]. E-cigarettes are expected to be regulated in the UK through European legislation by 2016 and a ban on sales to under 18s is expected to precede this in England [13,14]. At present, however, they are governed only through general product safety regulations with no specific sale or marketing restrictions. Trading Standards is the body responsible for enforcing regulations on the sale of consumer goods in the UK, including sales of age-restricted products. To inform their work in North West England, Trading Standards run biennial school surveys on alcohol and tobacco-related behaviours among 14-17 year old students, with the legal purchase age for both these substances being 18 years. With growing concerns around the exposure of children to unregulated e-cigarettes, a question was added to the 2013 survey asking students if they had ever bought or tried e-cigarettes. This question offers the opportunity to identify levels of e-cigarettes access among children under 18 years and the characteristics of those that are most likely to be accessing them; with preventing child access to e-cigarettes being a key focus of legislative approaches to e-cigarettes not only in the UK but internationally. Thus, here we explore associations between e-cigarette access, student demographics, and their patterns of tobacco and alcohol use.

## Methods

The study used data from the 5^th^ iteration of the Trading Standards North West Alcohol and Tobacco Survey conducted among 14-17 year olds in schools in North West England. The survey consists of closed, self-completed questions including: age, gender, alcohol consumption (drinking frequency, binge drinking frequency, drink types consumed, drinking location, drinking to get drunk) smoking behaviours (smoking status, age of first smoking), alcohol and tobacco access methods, parental smoking, and involvement in violence when drunk [15-17]. The question on e-cigarette access asked students “have you ever tried or purchased e-cigarettes”.

As with previous survey rounds, the questionnaire was made available to secondary schools across North West England through local authority Trading Standards departments [15-17]. Within schools, the questionnaire was delivered to students by teachers during normal school lessons between January and April 2013. Students self-completed the questionnaire voluntarily and anonymously. Compliance was not recorded by schools with the sample not intended to be representative of North West school children, but rather to provide a broad sample of students from a range of community types. A total of 114 schools participated, with representation from schools in all 23 upper tier local authorities in the region. The initial survey in 2005 was approved by the Trading Standards North West Executive Committee and schools participate on a voluntary basis under their own approval systems. Ethical approval to analyse the survey data for this study was obtained from Liverpool John Moores University Research Ethics Committee.

The total dataset included 18,233 participants aged 14-17 years. Cases were excluded if they were missing data on gender (n = 144), e-cigarette access (n = 602), or if questionnaires were spoiled (n = 23; e.g. unrealistic answers). The Index of Multiple Deprivation (IMD) 2010 [18] was used to assign participants to an ecological quintile of deprivation using a three stage process. Where full postcode was available (n = 8,676 cases), these were mapped directly to a Lower Super Output Area (English geographical areas with an average population size of 1,500 [19]) for which an IMD score is routinely calculated at a national level. Where only partial postcode was provided, those that spanned more than one LSOA were assigned to the LSOA that contained the majority of postcodes possible within the partial postcode (n = 2,926 cases). Where no postcode information was available, students were allocated to an LSOA based on school postcode; a proxy measure used in previous studies (n = 5,844 cases) [15-17]. Students were assigned to a national quintile of deprivation based on the IMD score of their allocated LSOA (1 = most affluent, 5 = most deprived). Students for whom no marker of deprivation was available were excluded from analyses (n = 1,253 cases). Thus, the final sample was 16,193.

Analysis was undertaken in SPSS (v18) using chi squared and logistic regression to control for confounding factors when examining associations between e-cigarette access and student demographics, smoking and drinking behaviours. Hierarchical logistic regression was used with school in the first block to control for clustering of students within schools, with the exception of analysis of regular smokers where sample sizes in individual schools were insufficient. E-cigarette access was identified through a ‘yes’ response to the question *Have you ever bought or tried electronic cigarettes?* Smoking status was established through a question asking students which of a range of options best described them (Table 1), with response options for smokers including *I only smoke when drinking alcohol* and increasing levels of regular smoking from <5 to >20 per day. Those smoking <5 a day were categorised as light regular smokers and those smoking at higher levels as heavy regular smokers. Ex-smokers were identified through the option ‘I used to smoke but have given up’. Alcohol consumption frequency was identified by the question *How often would you say you drink alcohol?* and binge drinking through the question *How often would you say that you drink five or more alcoholic drinks on one occasion?* (response options: never, <once a month, 1-3 times a month, once a week, twice a week, 3-6 times a week, every day). We categorised students into five drinker types: non-drinkers: occasional moderate drinkers (drink < once a week, never binge); regular moderate drinkers (drink > =once a week, never binge); occasional drinkers that binge (drink < once a week, binge at any frequency); and regular drinkers that binge (drink alcohol > =once a week, binge at any frequency). Students were identified as users of specific drink types (e.g. spirits) if they reported consuming at least one of that drink type in a typical week.

**Table 1:** **Associations between e-cigarette access and demographics, smoking and drinking status, and parental smoking**

		**Sample distribution**		**Accessed e-cigarettes**			
		**%**	**n**	**%**	**P** ^**a**^	**AOR (95% CIs)** ^**b**^	**P**
All		100.0	16193	19.2			
Gender	(Ref) Female	52.9	8569	17.7			
	Male	47.1	7624	20.8	<0.001	1.64 (1.47-1.82)	<0.001
Age	(Ref) 14	20.5	3318	16.3			
	15	45.4	7350	17.9			
	16	31.7	5141	22.1			
	17	2.4	384	28.4	<0.001		ns
Deprivation quintile	(least deprived, Ref) 1	12.5	2022	14.4			
	2	15.1	2447	16.4			
	3	15.8	2555	17.9			
	4	18.7	3035	21.8			
	(most deprived) 5	37.9	6134	21.0	<0.001		ns
Smoking status	(Ref) Never smoked	61.2	9699	4.9			<0.001
	Tried but didn’t like it	19.4	3082	22.6		5.10 (4.44-5.86)	<0.001
	Ex-smoker	5.1	803	50.7		17.94 (14.87-21.63)	<0.001
	Smoke when drinking	5.1	805	43.4		14.16 (11.62-17.26)	<0.001
	Regular light smoker	2.9	457	67.2		36.55 (28.64-46.64)	<0.001
	Regular heavy smoker	6.4	1012	75.8	<0.001	50.28 (40.97-61.71)	<0.001
Parent/guardian smokes	(Ref) No	59.4	9435	13.2			
	Yes	40.6	6454	27.4	<0.001	1.53 (1.37-1.70)	<0.001
Drinking status	(Ref) Non-drinker	31.7	5067	9.3			<0.001
	Occasional moderate	13.2	2115	8.2		0.96 (0.77-1.19)	0.719
	Regular moderate	1.1	175	13.7		0.91 (0.53-1.57)	0.744
	Occasional, binge	38.0	6066	21.8		1.46 (1.26-1.69)	<0.001
	Regular, binge	15.9	2544	41.9	<0.001	1.89 (1.59-2.24)	<0.001

^a^Chi squared analysis; ^b^Hierarchichal backward conditional logistic regression including all variables shown with school entered in the first block, included sample n = 15,400. AOR = adjusted odds ratio; CIs = confidence intervals; Ref = reference category; ns = not significant.

## Results

The demographic breakdown of the sample is shown in Table 1. One in five respondents (19.2%) reported having accessed e-cigarettes, with access being higher in males than in females and increasing with age and deprivation (Table 1). Levels of e-cigarette access increased from 4.9% of those who had never smoked cigarettes, to half (50.7%) of ex-smokers and over two thirds of regular smokers (67.2% light regular smokers, 75.8% heavy regular smokers, P < 0.001; Table 1). Thus, across all students who had accessed e-cigarettes, 35.8% were regular smokers, 11.6% only smoked when drinking, 13.6% were ex-smokers, 23.3% had tried smoking but didn’t like it, and 15.8% had never smoked. Teenagers with a parent/guardian who smoked were more likely to have accessed e-cigarettes than those with non-smoking parents/guardians (Table 1). E-cigarette access also showed a strong relationship with alcohol use. Students who drank alcohol at any level were significantly more likely to have accessed e-cigarettes than non-drinkers (23.7% v 9.3%, P < 0.001), with prevalence rising from less than one in ten in non-drinkers or occasional moderate drinkers to 41.9% in regular drinkers that binge (Table 1).

The relationship between e-cigarette access and male gender remained after adjusting for confounders through logistic regression (Table 1). However, relationships between e-cigarettes and age and deprivation were no longer significant. Any smoking experience was associated with e-cigarette access, with adjusted odds ratios (AORs) rising to 50.28 in regular heavy smokers. An independent relationship also remained between e-cigarette access and having parents/guardians that smoke. Compared with non-drinkers, binge drinkers had increased odds of e-cigarette access, with AORs rising from 1.46 for occasional drinkers to 1.89 for those drinking weekly or more.

Among regular smokers, e-cigarette access increased with age and was higher in males and those with parents/guardians who smoked (Table 2). Prevalence was also higher in smokers who got their cigarettes from parents or from friends/family aged over 18 years. After controlling for confounding factors, e-cigarette access in regular smokers remained associated with older age, being male, heavy smoking and having parents/guardians that smoked. However, only obtaining cigarettes from friends/family aged over 18 was associated with e-cigarette access (Table 2).

**Table 2:** **Associations between e-cigarette involvement, demographics, and smoking and drinking behaviours in regular smokers**

		**Sample distribution**		**Accessed e-cigarettes**			
		**%**	**n**	**%**	**P** ^**a**^	**AOR (95% CIs)** ^**b**^	**P**
Gender	(Ref) Female	54.3	797	70.5			
	Male	45.7	672	76.2	0.015	1.36 (1.04-1.80)	0.027
Age	(Ref) 14	15.9	234	63.7			0.003
	15	40.9	601	72.5		1.65 (1.12-2.41)	0.011
	16	38.5	565	77.0		2.10 (1.42-3.11)	<0.001
	17	4.7	69	78.3	0.001	2.21 (0.95-5.13)	0.066
Deprivation quintile	(least deprived; Ref) 1	7.4	109	71.6			
	2	10.7	157	71.3			
	3	15.8	232	76.3			
	4	22.0	323	73.1			
	(most deprived) 5	44.1	648	72.7	0.802		ns
Smoking status	(Ref) Light smoker	31.1	457	67.2			
	Heavy smoker	68.9	1012	75.8	0.001	1.56 (1.19-2.06)	0.001
Smoked < age 13	(Ref) No	63.1	806	74.2			
	Yes	36.9	472	74.4	0.946		
Parents/guardians smoke	(Ref) No	31.7	456	66.9			
	Yes	68.3	983	75.8	<0.001	1.48 (1.12-1.96)	0.007
Conventional cigarette access methods^c^							
Get from parents	(Ref) No	73.0	1006	72.1			
	Yes	27.0	372	77.7	0.036		ns
Get from friends/family under 18	(Ref) No	78.8	1086	74.2			
	Yes	21.2	292	71.2	0.304		ns
Get from friends/family over 18	(Ref) No	62.4	860	71.3			
	Yes	37.6	518	77.4	0.012	1.37 (1.03-1.82)	0.028
Buy from shops	(Ref) No	43.7	602	72.3			
	Yes	56.3	776	74.6	0.326		ns
Buy from street sellers etc*	(Ref) No	79.8	1099	73.5			
	Yes	20.2	279	73.8	0.915		ns
Drinking status	(Ref) non-drinker	7.3	105	79.0			
	Occasional moderate	3.0	44	65.9			
	Regular moderate	0.7	10	70.0			
	Occasional, binge	37.2	539	73.1			
	Regular, binge	51.8	749	72.8	0.535		ns

^a^Chi squared analysis; ^b^Backward conditional logistic regression including all variables shown, included sample n = 1,185. ^c^Students were instructed to tick all that applied. AOR = adjusted odds ratio; CIs = confidence intervals; Ref = reference category; ns = not significant.

*Buy from street sellers/neighbours/private house/vans.

In teenagers who had never smoked conventional cigarettes, e-cigarette access was higher in males, increased with deprivation and was elevated in those whose parents/guardians smoked (Table 3). E-cigarette access was strongly associated with alcohol use, with prevalence ranging from 1.8% in regular moderate drinkers up to 11.5% in regular drinkers who binge. This relationship remained in multivariate analysis, with odds of e-cigarette access being 4.59 in non-smokers that regularly drink and binge compared with those that do not drink (Table 3). Multivariate analysis also found reduced odds of e-cigarette access in 15 and 16 year olds compared with 14 year olds.

**Table 3:** **Associations between e-cigarette involvement, demographics and drinking behaviours in school children that have never smoked**

		**Sample distribution**		**Accessed e-cigarettes**			
		**%**	**n**	**%**	**P** ^**a**^	**AOR (95%CIs)** ^**b**^	**P**
Gender	(Ref) Female	50.8	4931	3.4			
	Male	49.2	4768	6.4	<0.001	1.96 (1.59-2.42)	<0.001
Age	(Ref) 14	23.1	2238	5.3			0.038
	15	46.7	4532	4.5		0.72 (0.55-0.94)	0.011
	16	28.4	2752	5.1		0.72 (0.53-0.98)	0.038
	17	1.8	177	6.2	0.331	1.31 (0.63-2.75)	0.470
Deprivation quintile	(least; Ref) 1	14.1	1367	3.9			ns
	2	16.3	1579	3.8			
	3	15.8	1533	4.4			
	4	18.1	1753	5.4			
	(most) 5	35.7	3467	5.7	0.007		
Parents/guardians smoke	(Ref) No	67.1	6422	4.0			
	Yes	32.9	3143	6.5	<0.001	1.57 (1.28-1.93)	<0.001
Alcohol use	(Ref) Non-drinker	43.6	4180	3.6			<0.001
	Occasional moderate	17.5	1679	3.6		1.38 (1.01-1.90)	0.045
	Regular moderate	1.1	110	1.8		0.57 (0.14-2.40)	0.446
	Occasional, binge	30.4	2915	5.9		2.39 (1.87-3.06)	<0.001
	Regular, binge	7.3	704	11.5	<0.001	4.59 (3.34-6.29)	<0.001

^a^Chi squared analysis; ^b^Hierarchical backward conditional logistic regression including all variables shown, included sample n = 9,458. AOR = adjusted odds ratio; CIs = confidence intervals; Ref = reference category; ns = not significant.

Limiting data to students who drink alcohol, bivariate relationships were seen between e-cigarette access and binge drinking, regular drinking and drinking alcohol in public places (e.g. in parks, streets; Table 4). Prevalence also increased in teenagers who purchased their own alcohol (from on- or off-licensed premises), took it from parents without consent, got it from friends/family either under or over 18, and got adults outside shops to buy it for them. However, it was reduced in those who were given or bought alcohol by parents (Table 4). E-cigarette access was higher in drinkers who agreed with the statement *I only drink alcohol to get drunk* and in those who reported having been involved in violence when drunk (Table 4). These strong relationships remained in multivariate relationships (Table 4). Independent relationships were also identified between e-cigarette access and: binge drinking; buying own alcohol from shops or supermarkets; getting alcohol from friends/family over aged 18; and getting alcohol from adults outside shops.

**Table 4:** **Associations between e-cigarette access and alcohol-related behaviours in drinkers**

		**Sample distribution**		**Accessed e-cigarettes**			
		**%**	**n**	**%**	**P** ^**a**^	**AOR (95% CIs)** ^**b**^	**P**
Binge drink	No (Ref)	21.0	2293	8.6			
	Yes	79.0	8630	27.8	<0.001	1.48 (1.20-1.82)	<0.001
Drinking frequency	<weekly (Ref)	75.1	8310	18.3			
	> = weekly	24.9	2758	40.0	<0.001		
Drink outside	(Ref) No	86.5	9604	20.3			
	Yes	13.5	1496	45.7	<0.001		
Alcohol access methods^c^							
Buy in pubs and clubs	(Ref) No	92.7	9851	22.4			
	Yes	7.3	780	41.5	<0.001		
Buy from off licences/supermarkets	(Ref) No	88.8	9436	20.6			
	Yes	11.2	1195	49.2	<0.001	1.29 (1.08-1.55)	0.005
Parents/guard ians give/buy	(Ref) No	42.9	4566	31.8			
	Yes	57.1	6065	17.7	<0.001		
Take from parents/guardians without consent	(Ref) No	93.9	9985	22.9			
	Yes	6.1	646	37.0	<0.001		
Friends/family under 18	(Ref) No	88.9	9448	22.7			
	Yes	11.1	1183	32.5	<0.001		
Friends/family over 18	(Ref) No	53.7	5710	20.8			
	Yes	46.3	4921	27.2	<0.001	1.24 (1.09-1.40)	0.001
Get adults outside shops to buy for me	(Ref) No	89.9	9556	21.0			
	Yes	10.1	1075	48.9	<0.001	1.28 (1.06-1.54)	0.010
I only drink alcohol to get drunk	(Ref) No	64.4	6934	15.8			
	Yes	35.6	3833	37.5	<0.001	1.27 (1.11-1.45)	0.001
Been violent or in a fight when drunk	(Ref) No	83.1	9029	17.1			
	Yes	16.9	1836	54.8	<0.001	1.78 (1.52-2.08)	<0.001

^a^Chi squared analysis; ^b^Hierarchical backward conditional logistic regression, included sample n = 9,651: the variables gender, age, deprivation quintile, smoking status and parent/guardian smoking were also included in the model; male gender, any smoking experience and parental smoking were significantly associated with e-cigarette access. ^c^Students were instructed to tick all that applied. AOR = adjusted odds ratio; CIs = confidence intervals; Ref = reference category; ns = not significant.

A total of 6,323 drinkers provided details on the drink types they consumed in a normal week. Spirits were the most common type of drink consumed (63.1%), followed by lager (52.7%), alcopops (51.6%), bottles/cans of cider (49.4%), wine (42.9%) and large value bottles of cider (31.5%). A logistic regression model using all variables in Table 4 plus drink type variables found that consumption of spirits (AOR 1.32 [1.10-1.60], P = 0.004) was independently associated with e-cigarette access.

## Discussion

As public health debate on the pros and cons of e-cigarettes continues, moves to regulate their promotion and sale are increasingly being proposed and adopted by governments across the world [6]. A key feature of such regulation is preventing child access to e-cigarettes. Thus, understanding the extent of e-cigarette access by young people and the characteristics of those that access them will be crucial in informing prevention and control strategies. Using a sample of over 16,000 14-17 year old school students in North West England, we found that almost one in five had either tried or purchased e-cigarettes. Such rapid penetration into teenage culture of what is essentially a new drug use option is without precedent. As with findings from studies elsewhere [7-9], e-cigarette access was most common in students who smoked conventional cigarettes, particularly those who smoked in greater quantities. Thus, 67.2% of light smokers (<5 per day) and 75.8% of heavier smokers reported having accessed e-cigarettes. These figures are far higher than those for e-cigarette use by adolescent smokers reported in previous studies (e.g. France, 33.4% [8], Korea, 36.6% [9]), and this will in part reflect the different age ranges and broader question asked in our study. However, findings are consistent with other recent studies reporting high levels of e-cigarette access among tobacco smokers (e.g. USA [20]), and this may indicate the rapid expansion in promotion [10] and reducing price [21] of e-cigarettes that means they are widely visible and easily available to teenagers with an interest in smoking.

A key public health concern with e-cigarettes is their potential to recruit children to nicotine dependence. In adults, e-cigarettes are typically used by smokers to help them reduce or quit tobacco use and uptake levels among non-smokers are thought to be very low (typically <1%) [22-25]. Here, however, almost one in twenty (4.9%) teenagers who had never smoked conventional cigarettes reported having accessed e-cigarettes. In fact, although ex-smokers had greater odds of e-cigarette access than never smokers, never smokers accounted for a much larger proportion of the sample (61.2% v 5.1% for ex-smokers) and therefore a larger proportion of those reporting e-cigarette access (15.8%) than ex-smokers (13.6%). Among never-smokers, odds of having accessed e-cigarettes were greater in 14 year olds than in those aged 15 or 16 years. This likely reflects the fact that many young people who are inclined towards smoking will have already tried cigarettes by the age of 15. For individuals who have never used a substance, however, accessing that substance is a first step to initiating use. Further, for conventional tobacco products, perceived ease of accessibility has been found to increase adolescents’ risk of smoking uptake [26].

In the UK, as in many other countries, today’s adolescents have grown up with a strong ‘smoking kills’ message and reducing social acceptance of smoking. Smoking prevention education is delivered in schools from an early age, tobacco advertising is banned, tobacco packaging carries strong visual health warnings and smoke-free legislation prohibits smoking in virtually all enclosed work and public spaces. As tobacco control and smoking prevention activity has increased, smoking prevalence in children has reduced, with 23% of 11-15 year olds in England in 2012 having ever tried smoking compared with 49% in 1996 [27]. However, adolescence is a period of experimentation and while anti-smoking efforts may have deterred many teenagers from trying tobacco, the marketing of e-cigarettes as a healthy alternative may proffer a viable new method for them to experience the nicotine ‘hit’ without the perceived harmful impacts of tobacco. For other products, health claims have been found to produce a ‘halo effect’ that not only encourages purchasing, but also reduces consumers’ information-seeking (e.g. food and nutritional information [28]). Specifically for teenagers, glamorisation of e-cigarettes in advertising, celebrity endorsement and the range of attractive designs and flavourings available are likely to be furthering their appeal [3]. Here, almost a quarter of teenagers that had accessed e-cigarettes had tried smoking conventional cigarettes but not liked them. Although we cannot determine whether this experience occurred before or after accessing e-cigarettes, it is likely that flavourings make e-cigarettes an attractive option to teenagers who would otherwise be put off conventional cigarettes by their taste.

A key finding from our study was the association between alcohol consumption and e-cigarette access. Even after controlling for smoking behaviours, teenagers who drink regularly and binge drink were significantly more likely to have accessed e-cigarettes (Table 1). This association was particularly strong in those that had never smoked. Thus, over one in ten never-smokers who drank regularly and binged had accessed e-cigarettes, with their odds of e-cigarette access more than four times those of never-smokers who did not drink. Among all drinkers, e-cigarette access was associated with binge drinking, drinking to get drunk, involvement in violence after drinking and consumption of spirits; a drink type that has been associated with alcohol-related harm in previous studies [16]. These findings suggest that teenagers that access e-cigarettes are those that are most vulnerable to other forms of substance use and risk-taking behaviours, and that e-cigarettes are rapidly being added to at-risk teenagers’ substance using repertoires.

The high prevalence of e-cigarette access amongst teenagers in our study, and particularly their use among those that have never smoked conventional cigarettes, highlights the urgent need for age restrictions on the promotion and sale of e-cigarettes. With such restrictions increasingly being introduced, understanding how teenagers that access e-cigarettes are able to access other age-restricted products (i.e. cigarettes and alcohol) will support enforcement work. Among smokers, obtaining cigarettes from friends or family over the age of 18 was independently associated with e-cigarette access. Friends and family are also key sources of e-cigarettes for teenagers [29]. Among drinkers, teenagers who bought alcohol themselves from off-licensed premises and those who asked adults outside shops to purchase alcohol for them (known as proxy purchasing) also had increased odds of e-cigarette access. Both these methods represent mechanisms by which minors access alcohol illicitly without the knowledge or supervision of parents or guardians, and both have been associated with risky alcohol consumption and alcohol-related harms in previous studies [15,16]. Their association with e-cigarettes suggests that teenagers who access e-cigarettes are already familiar with strategies to bypass age legislation on restricted products.

Across all analyses conducted in our study, having a parent or guardian who smokes was one of the strongest predictors of e-cigarette access. Associations between parental and child smoking are widely reported [30], and are thought to operate through both genetic and environmental influences. Similar relationships may exist for propensity to experiment with e-cigarettes. The Trading Standards survey did not ask students whether or not their parents used e-cigarettes, yet the availability of e-cigarettes in the home may be an important consideration for future interventions. Although the content of e-cigarettes varies [31], single cartridges typically contain several hundred ‘puffs’ and unguarded, could easily be ‘shared’ by children without the adult users’ knowledge. The involvement of parents along with schools in work to address e-cigarette use in children is likely to be particularly important as their lack of smoke and odour means that, unlike conventional cigarettes, they can easily be used in bedrooms or on school property without detection.

Like all cross-sectional surveys, this study had a number of limitations. Firstly the question on e-cigarettes included in the Trading Standards survey was specific to access, asking whether students had tried or bought e-cigarettes. However, preventing child access to e-cigarettes is the focus of current regulatory responses to e-cigarettes. While it is not possible to determine how many of those teenagers who accessed e-cigarettes had either bought or used them, it seems reasonable to assume that many teenagers who are motivated to purchase an e-cigarette would also be interested in trying it. The survey did not record any measure of e-cigarette access frequency nor of when e-cigarette access had occurred, and thus it was not possible to identify whether teenagers that reported both e-cigarette access and smoking had accessed e-cigarettes before or after conventional cigarettes. While school surveys can be limited by time availability in the classroom, results from this survey justify a greater focus on e-cigarettes in the next iteration of the survey, including questions on frequency of e-cigarette use and age of initiation. School participation was voluntary and compliance data were not recorded, thus selection bias cannot be ruled out. In the absence of residential postcode data, students were assigned to a quintile of deprivation on an ecological basis and while this may have meant some students were misallocated, the deprivation profile of the sample was generally consistent with that for the 14-17 year old population in the North West region (sample, quintile 1, 12.5%; 2, 15.1%; 3, 15.8%; 4, 18.7%; 5, 37.9%; North West, quintile 1, 15.6%; 2, 16.3%; 3, 15.6%; 4, 18.0%; 5 34.5%). Students that could not be assigned to a deprivation quintile were excluded from analysis and therefore represent additional potential bias in the final sample. As with all surveys of self-reported social behaviours, students’ may have under or over reported e-cigarette access, smoking and drinking behaviours due to factors including social desirability, poor recall or lack of knowledge. Finally, while little data are available on the regional distribution of e-cigarette use among either children or adults in England, prevalence of tobacco smoking tends to be higher in the North than in the South [32]. Thus findings should not be considered representative of all 14-17 year olds in England or the North West region.

## Conclusions

Identifying which children are accessing e-cigarettes is crucial for targeting health information and understanding which young people may be most vulnerable as sales of e-cigarettes become age restricted. Our study suggests that a substantial number of teenagers are accessing e-cigarettes, including those who have never smoked conventional tobacco products. However, those most likely to access e-cigarettes are those who engage in other substance-related risk behaviours including regular smoking, binge drinking, drinking to get drunk and alcohol-related violence. Thus, findings appear more consistent with teenagers viewing e-cigarettes as a recreational substance rather than a smoking cessation tool. E-cigarette access is also associated with specific alcohol access patterns, including self-purchase of alcohol in off-licensed premises and the recruitment of proxy purchasers from outside such premises. Thus, high risk teenagers that access e-cigarettes are likely to already be familiar with mechanisms for avoiding age restrictions on substances. In particular, findings highlight the urgent need for controls on e-cigarette sales to children. The longer such controls are delayed, the greater the number of children likely to want to access e-cigarettes illicitly once a ban on sales to children is imposed.
